# Endothelial Progenitor Cells in Coronary Artery Disease: From Bench to Bedside

**DOI:** 10.1093/stcltm/szac010

**Published:** 2022-04-01

**Authors:** Francesco Pelliccia, Marco Zimarino, Giuseppe De Luca, Nicola Viceconte, Gaetano Tanzilli, Raffaele De Caterina

**Affiliations:** 1 Department of Cardiovascular Sciences, Sapienza University, Rome, Italy; 2 Institute of Cardiology, “G. d’Annunzio” University, Chieti, Italy; 3 Cath Lab, SS. Annunziata Hospital, Chieti, Italy; 4 Division of Cardiology, Azienda Ospedaliero-Universitaria Maggiore della Carità, Università del Piemonte Orientale, Novara, Italy; 5 University of Pisa and Pisa University Hospital, Pisa, Italy

**Keywords:** coronary artery disease, endothelial progenitor cells, percutaneous coronary intervention, prognosis

## Abstract

Endothelial progenitor cells (EPCs) are a heterogeneous group of cells present in peripheral blood at various stages of endothelial differentiation. EPCs have been extensively investigated in patients with coronary artery disease (CAD), with controversial findings both on their role in atherosclerosis progression and in the process of neointimal growth after a percutaneous coronary intervention (PCI). Despite nearly 2 decades of experimental and clinical investigations, however, the significance of EPCs in clinical practice remains unclear and poorly understood. This review provides an update on the role of EPCs in the most common clinical scenarios that are experienced by cardiologists managing patients with CAD. We here summarize the main findings on the association of EPCs with cardiovascular risk factors, coronary atherosclerosis, and myocardial ischemia. We then discuss the potential effects of EPCs in post-PCI in-stent restenosis, as well as most recent findings with EPC-coated stents. Based on the mounting evidence of the relationship between levels of EPCs and several different adverse cardiovascular events, EPCs are emerging as novel predictive biomarkers of long-term outcomes in patients with CAD.

## Graphical Abstract

**Figure:**
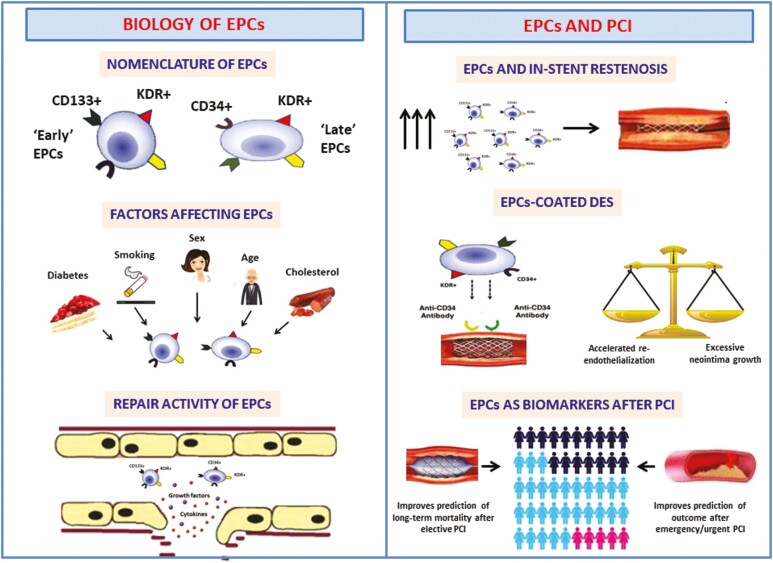
Endothelial progenitor cells (EPCs) can be differentiated based on surface antigens, are affected by several cardiovascular risk factors, and play a role in vascular repair. After percutaneous coronary intervention, EPCs are involved in the process of in-stent restenosis and might predict the outcome. EPCs-coated drug-eluting stents induce accelerated re- endothelialization but can favor excessive neointima growth.

**Lessons Learned**

- Endothelial progenitor cells (EPCs) are a heterogeneous group of cells present in peripheral blood at various stages of endothelial differentiation that play a role in atherosclerosis progression.
- Assessment of circulating EPCs might improve risk stratification in coronary artery disease beyond the traditional assessment model.
- EPCs might modulate neointimal growth after percutaneous coronary intervention (PCI) thus having a potential role in post-PCI restenosis and EPCs-coated stents

**Significance Statement**

In recent years, there has been emerging evidence that circulating levels of endothelial progenitor cells (EPCs) have the ability to provide information on the atherosclerotic burden, are associated with in-stent restenosis after a percutaneous coronary intervention, and might predict long-term outcome in multiple cardiovascular conditions. These novel findings have led to a paradigm shift in the understanding of EPCs, which are now broadly considered as biomarkers of cardiac disease. A better understanding of their biological properties is crucial and should be of interest in clinical cardiology.

## Introduction

Endothelial progenitor cells (EPCs) are a heterogeneous group of cells present in peripheral blood at various stages of differentiation^1^ that have been extensively investigated in patients with coronary artery disease (CAD).^2^ Despite nearly 2 decades of experimental and clinical investigations, however, the translation of basic research into clinical practice has been dampened by unresolved questions around EPC definition and functions that both remain poorly understood by cardiologists managing patients with CAD.^3-5^

Soon after their early identification,^6^ pioneering investigations showed mobilization of these cells during angiogenesis, leading to the concept that EPCs might play a major role in vascular repair.^1^ However, the subsequent evidence that EPCs do not home at sites of developing atherosclerotic lesions led to the conclusion that these cells do not have a direct regenerative role.^3^ These findings have been coupled with the negative results of a number of clinical trials that failed to detect the engraftment of EPCs in areas of infarcted myocardium.^7^ In recent years, there has been emerging evidence that circulating levels of EPCs have rather the ability to provide information on the atherosclerotic burden, are associated with in-stent restenosis after a percutaneous coronary intervention (PCI), and might predict long-term outcome in multiple cardiovascular conditions. These novel findings have led to a paradigm shift in our understanding of EPCs, which are now broadly considered as biomarkers of cardiac disease.^8^ As a consequence, a better understanding of their biological properties is crucial and should be of interest also in clinical cardiology. The aim of this review is, therefore, to provide an update on the role and significance of EPCs in the most common clinical scenarios of patients with CAD. The manuscript recaps the biology of EPCs, their association with cardiovascular risk factors, coronary atherosclerosis, and myocardial ischemia, their potential role in-stent restenosis, the most recent findings with EPCs-coated stents, and finally the mounting evidence of their relationship with long-term adverse cardiovascular events.

## Biology of EPCs

### Origin and Differentiation of EPCs

EPCs were primarily isolated from peripheral blood mononuclear cells by Asahara et al.^6^ These authors showed that CD34^+^ hematopoietic progenitor cells from adults can differentiate into endothelial cells. Later on, a number of experimental studies found that these cells can increase angiogenesis in ischemic tissues.^1^ Accordingly, EPCs have long been said to have angiogenic potential that is acquired from the bone marrow and to form an important framework involved in the repair of endothelial damage.^1^ More recently, however, Fujisawa et al have shown that endogenous neovascularization in the heart is driven by tissue-resident progenitors,^7^ thus confirming previous evidence that endothelial cells do not derive from the bone marrow but rather arise from alternative niches in the vessel wall.^9^ Regardless of their origin, the consensus is that EPCs have an endothelial characterization and a vascular tropism, and are indicative of the endogenous regenerative capacity of the vascular system.^10^

### Identification and Characterization of EPCs

**Figure 1.**
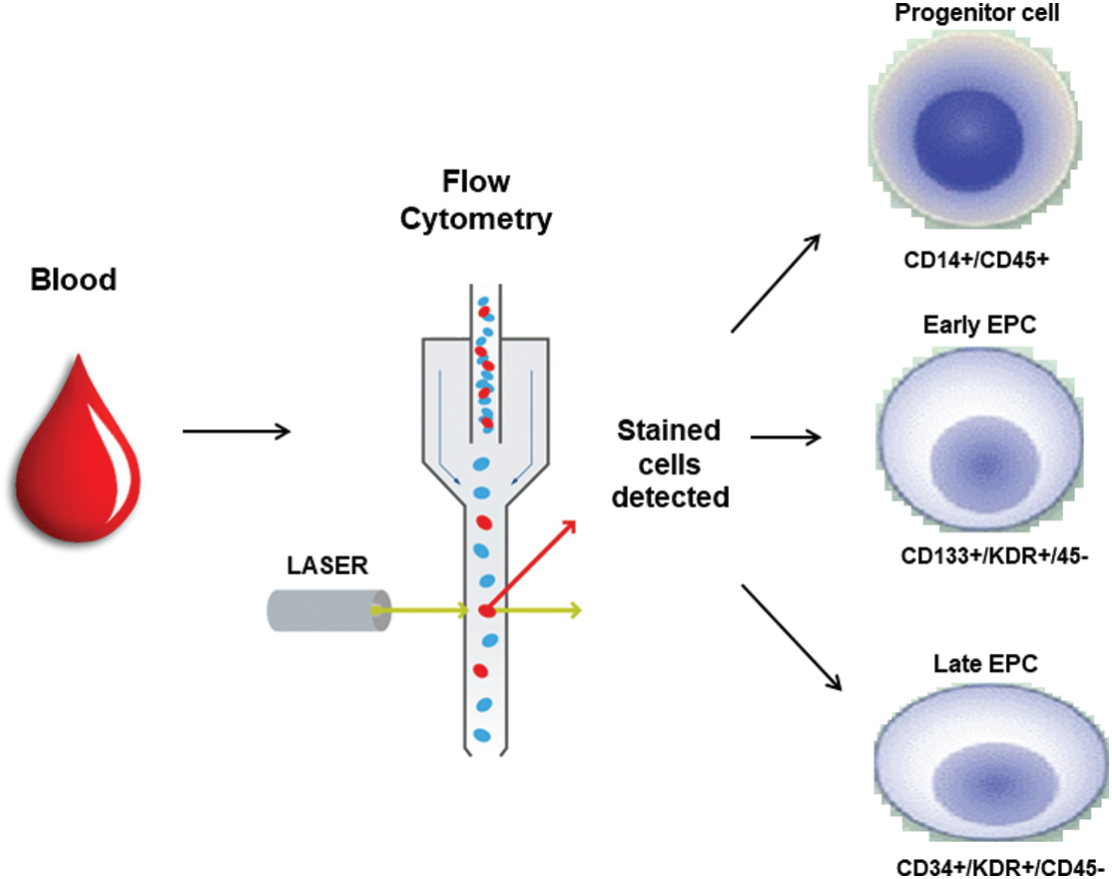
Expression of surface markers during the differentiation of the EPCs. There is a continuous shift in expression of stem and endothelial surface markers during the differentiation of EPCs. ‘Early’ EPCs are generally identified by expression of CD133 (an early hematopoietic stem cell marker) and KDR (an endothelial marker). Circulating “late” EPCs are more mature cells and are characterized by high expression of CD34 and KDR but decreased expression of CD133. Mature endothelial cells are terminally differentiated cells characterized by negative expression of CD34, CD133, and CD45, and positive expression of KDR, CD14, CD31, CD146, VE-cadherin, eNOS, and vWF. eNOS, endothelial nitric oxide synthase; EPCs, endothelial progenitor cells; KDR, kinase insert domain receptor; vWF, von Willebrand factor.

**Figure 2.**
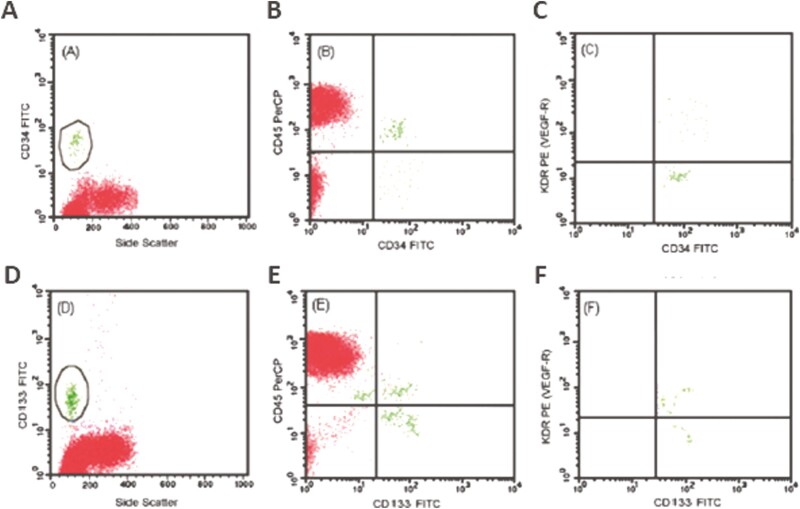
Representative flow cytograms of major subtypes of EPCs. After identifying peripheral blood progenitor cells, CD34^+^ cells and CD133^+^ cells are gated in the mononuclear cell fraction (left panels **A** and **D**). After separate gating to exclude hematopoietic cells expressing the CD45 antigen (middle panels **B** and **E**), CD34^+^/KDR^+^/CD45^−^ cells are identified as CD34^+^/CD45^−^ cells positive for KDR (**C**), and CD133^+^/KDR^+^/CD45^−^ cells are identified as CD133^+^/CD45^−^ cells positive for KDR (**F**). EPCs, endothelial progenitor cells; KDR, kinase insert domain receptor.

EPCs are present in peripheral blood at different stages of endothelial differentiation. They are a small fraction (between 0.01% and 0.3%) of blood mononuclear cells. There are 2 different methodological approaches to identify and characterize EPCs: flow cytometry and cell culture methods. Currently, flow cytometry is the technique of choice because the direct isolation of cell populations by using surface antigens has the advantage of selecting defined populations of cells without the limitations of ex vivo manipulation  (Fig. 1).^11^ With flow cytometry, EPCs are generally identified from blood cells through the expression of several surface markers (Fig. 2). These are proteins and carbohydrates attached to the cell membrane, which allow cell identification by providing a specific target. Unfortunately, many surface markers are shared either by hematopoietic stem cells or by adult endothelial cells, and cannot, therefore, be used in isolation to correctly identify progenitor cells.^12^ Examples of ambivalent markers are platelet endothelial cell adhesion molecule-1, melanoma cell adhesion molecule (CD146), vascular endothelial cadherin (CD144), endothelial nitric oxide synthase, and von Willebrand factor. For this reason, EPCs are currently defined as cells positive for both a stem cell marker (ie, the CD34 and CD133 antigens) and an endothelial protein such as Kinase insert domain receptor (KDR, a type IV receptor with tyrosine kinase activity—also known as vascular endothelial growth factor [VEGF] receptor 2). CD34 is an antigen not exclusively expressed on hematopoietic stem cells, but also on mature endothelial cells,^13^ while CD133 is a highly conserved antigen with unknown biological activity, expressed on hematopoietic stem cells but absent on mature endothelial and monocytic cells.^13^ Thus, CD133^+^KDR^+^ cells more likely reflect immature progenitor cells (“early” EPCs) and CD34^+^KDR^+^ may represent more mature cells (“late” EPCs). Both cell subtypes are now considered to have true endothelial progenitor capacity when they do not express the hematopoietic cell surface marker CD45 (Fig. 2).^10,12^

### Paracrine Activity of EPCs

**Figure 3.**
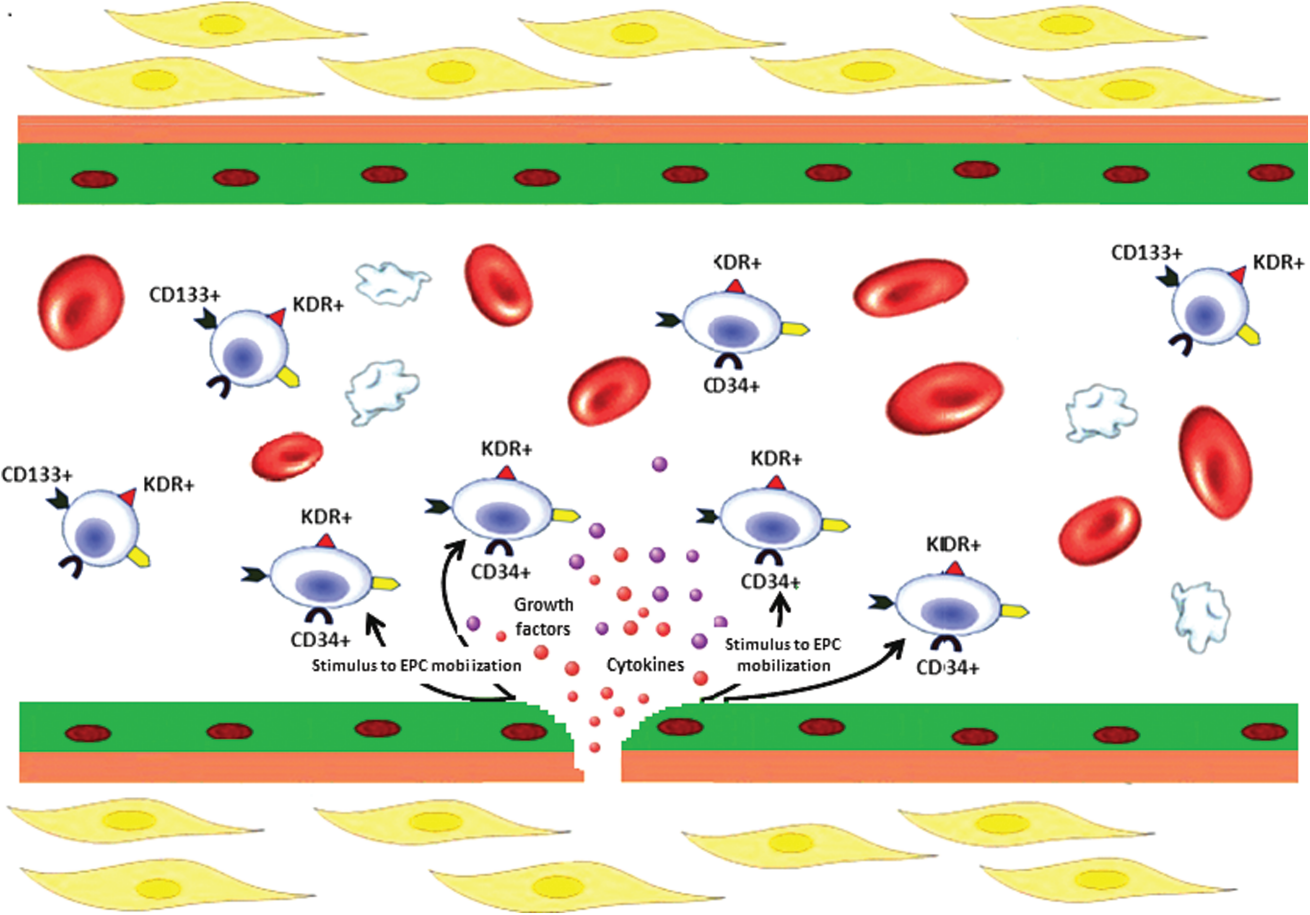
Paracrine activity of EPCs at the site of endothelial damage. When the endothelial layer is damaged, circulating EPCs are stimulated to act through paracrine mechanisms leading to the secretion of various cytokines and pro-angiogenic growth factors, such as VEGF, SDF-1, and NO. The paracrine signaling mediated by EPCs results in the production of an angiogenic microenvironment that stimulates the nearby endothelium to proliferate. EPCs, endothelial progenitor cells; SDF-1, stromal-derived factor-1; VEGF, vascular endothelial growth factor.

In recent years, it has become evident that EPCs do not support tissue repair through differentiation and incorporation into nascent vessels, but that EPC-derived paracrine signals play a pivotal role in orchestrating the repair processes in damaged tissues.^14^ EPCs are first mobilized into peripheral blood in response to chemo-attractants released by ischemic or damaged tissues,^15^ as well as in response to angiogenic factors released from atherosclerotic plaques.^16^ Of importance, a primary role in the transduction of mitotic and pro-survival signals of many growth factors and cytokines is played by 2 major signaling pathways, the phosphoinositide-3-kinase/serine/threonine-protein kinase,^17^ and the mitogen-activated protein kinases, mainly extracellular signal-regulated kinase.^18^ Although the way this complex mixture of factors modulates activities of target cells at the molecular level remains elusive, there is a general agreement that the paracrine signaling mediated by EPCs results in the production of an angiogenic microenvironment that stimulates the nearby endothelium to proliferate (Fig. 3),^11^ which in turn supports the concept that EPCs are novel biomarkers of cardiovascular health and disease.^8^

## EPCs and Coronary Atherosclerosis

### EPCs and Cardiovascular Risk Factors

Count and function of EPCs are affected by multiple factors associated with endothelial dysfunction, including age, sex, smoking, hypertension, diabetes mellitus, and dyslipidemia.^19^ Advancing age, one of the strongest determinants of cardiovascular risk, correlates inversely with EPCs.^20^ This is consistent with the experimental finding that reparative effects of EPCs decline with both aging and prolonged exposure to cardiovascular risk factors in a murine model of atherosclerosis.^21^ Sex is another major determinant of EPCs changes. The number of EPCs is greater in fertile female than in male,^22^ but a decline in circulating EPC is common in postmenopausal females.^23^ Similarly, a previous study showed that normal women had significantly higher absolute numbers of CD34^+^, CD133^+^, CD105^+^, and CD14^+^ cells than both normal men or women with CAD.^19^ These findings are in keeping with the observation that the lack of estrogen protection might yield to decreased endogenous endothelial repair capacity.^3^ Essential hypertension is an additional cause of lower levels of EPCs and impaired endothelial repair capacity.^5^ Cigarette smoking impairs EPCs and smoking cessation restores circulating EPCs in chronic smokers. ^24^Oxidized low-density lipoprotein induces EPC senescence as well.^25^ Last but not least, diabetes is one of the traditional risk factors most strongly associated with quantitative defects and functional impairment of stem cells, including EPCs.^3^ Overall, the strong association of EPCs with cardiovascular risk factors is in keeping with the emerging concept that EPCs can be regarded as novel biomarkers that reflect the integrity and repair capacity of the endothelium.^26^

### EPCs and Angiographically Assessed Coronary Atherosclerosis

EPCs have long been considered an important link for the well-recognized association between cardiovascular risk factors and coronary atherosclerosis. This concept has been originally supported by several investigations that assessed the relationship of EPC count with the extent of angiographically assessed CAD (Supplementary Table 1) .^27-36^ Interestingly, some studies reported a negative association between EPCs and the atherosclerotic burden,^27,28,30,31,33,35^ whereas others found an increased number of EPCs in patients with significant CAD.^29,32^ Such discordant findings can be explained at least partly by the influence that concomitant pathologic conditions have on circulating EPCs.^8^ Indeed, it is well established that multiple comorbidities such as diabetes mellitus, metabolic syndrome, aging, and smoking might diminish EPCs counts, which in turn might jeopardy the evaluation of the correlation with atherosclerosis. A further role might be played by heterogeneity in the definition of EPCs, which, unfortunately, have not been rigorously and uniformly assessed by most investigations.^4^ More recently, a neutral role for EPCs in atherosclerosis has been found in studies that have applied a validated methodology for the EPC quantification of multiple subtypes.^34,35^

Conflicting results have also emerged from studies exploring whether circulating EPCs play any role in the progression of CAD. Preliminary findings were in keeping with the hypothesis of pro-atherogenetic properties of EPCs,^37^ whereas later reports found that the EPCs were either unrelated^34^ or even lower in subjects with angiographic evidence of progression of CAD compared with controls.^38,39^ EPCs promote the development of collateral circulation through an angiogenesis-mediated mechanism over the course of days to weeks after the ischemic stimulus but have also a proatherogenic capacity over months to years.^40^ According to Epstein et al.,^41^ these ambivalent properties may account for a “Janus phenomenon,” as EPCs appear to have 2 faces, one looking forward (ie, collateral formation) and one looking backward (ie, atherogenesis), as the Roman deity Janus.

### EPCs and Myocardial Ischemia

Repetitive episodes of transient myocardial ischemia are associated with adaptive processes leading to neovascularization.^42^ Recently, Hammadah et al have shown that patients without ischemia had a 15% increase in CD34^+^ cells counts after exercise, whereas patients with myocardial ischemia had an 18% postexercise reduction.^43^ Interestingly, this latter EPC decrease was proportional to the magnitude of ischemia and to the change in circulating levels of a stromal-derived factor (SDF)-1α, a cytokine that stimulates homing of EPCs to the ischemic myocardium.

In patients with the acute coronary syndrome (ACS), EPCs seem, again, to play a Janus-type role. Experimental models have demonstrated the ambivalent effects of EPCs, which can be either protective—by promoting endothelial integrity—or detrimental—by inducing intra-plaque angiogenesis and abrupt plaque growth.^44^

In patients presenting with acute myocardial infarction (AMI), EPC counts are more than doubled compared to patients with stable CAD.^45,46^ The mobilization of EPCs starts within a few minutes after AMI, peaks after several days and normalizes within 60 days.^45^ The link between myocardial ischemia and EPCs appears to be mediated by inflammation. Ischemia and tissue injury trigger an acute inflammatory response with an upregulation of hypoxia-dependent factor (HIF)-1α that, in turn, stimulates the expression of SDF-1 and VEGF, leading to recruitment of EPCs to ischemic tissues.^47^ As previously described, this homing of EPCs to areas of ischemia stimulates angiogenesis through the paracrine secretion of angiogenic growth factors,^48^ with the tradeoff of accelerating atherosclerotic plaque formation and lesion size.^49^

## EPCs and PCI

### Acute Effects of PCI on EPCs

Stent deployment causes mechanical injury to the vessel wall that induces substantial local inflammation, stimulating vascular smooth muscle cell proliferation, and extracellular matrix depositions.^50^ Thomas et al demonstrated a fall in EPC levels 6 h after the procedure.^51^ Similarly, Montenegro et al more recently described a decrease in EPC counts after PCI in about two-thirds of the patients.^52^ These results are at variance from those of Bonello et al^53^ and Lee et al^54^ who observed post-procedural increases in EPCs. A possible explanation of these discordant findings can be found in the study of Gao et al, who demonstrated that elective PCI triggers a time-dependent mobilization of CD34^+^/KDR^+^ cells that closely correlates with the extent of the endothelial injury caused by the procedure.^55^

### EPCs and Stent Restenosis

Although there is a general consensus that EPCs are involved in post-PCI processes, studies assessing the relation of EPC with the subsequent occurrence of restenosis have yielded discordant results (Supplementary Table 2). ^34,39,52,53,56-61^

**Figure 4.**
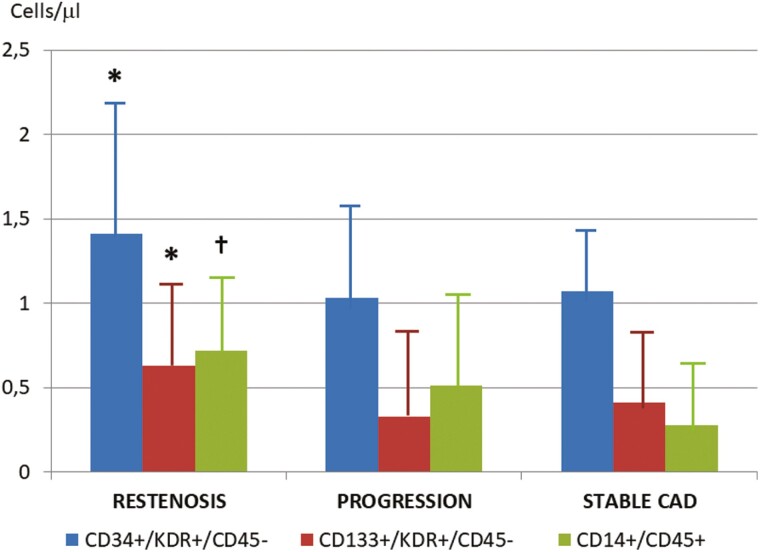
Endothelial progenitor cells and restenosis. Endothelial progenitor cells at the time of PCI in patients who had evidence at follow-up angiography of in-stent restenosis, progression of coronary atherosclerosis in non-stented segments, or unchanged coronary anatomy. Data are expressed as *n*, mean ± SD, or *n* (%). *P < .05 for comparison between restenosis versus progression and stable groups. †*P* < .05 for comparison between restenosis group versus stable group. Modified from Pelliccia  et al.^34^ PCI, percutaneous coronary intervention.

In the bare-metal stent era, the post-PCI increase of EPCs over baseline identified patients at higher risk of developing restenosis. Here the best discriminant parameter was identified as an augmented count of CD34^+^ cells .^56,57^ Similarly, Pelliccia et al found that patients who subsequently experienced in-stent restenosis had higher levels of circulating CD34^+^, CD133^+^, and CD14^+^ cells with respect to stable patients and controls, indicating that excessive intima proliferation and in-stent restenosis may occur particularly in those patients with the highest EPC levels at baseline (Fig. 4).^34^ These findings are consistent with experimental models demonstrating that CD14^+^/CD45^+^ monocyte-derived cells enhance neovascularization.^62^ Mixed results, however, have been reported in other investigations, reporting an association of neointimal hyperplasia with high^61^ or low EPC counts,^58,59^ or finding that restenosis was unrelated to EPCs.^53,60^

Number and function of EPCs seem to be significantly affected also by drug-eluting stent (DES).^63^ First-generation DES were shown to reduce both late lumen loss and CD34^+^ cell mobilization, thus suggesting that neointimal suppression is linked with impaired re-endothelialization. Similarly, using second-generation DES, the number of uncovered stent struts paralleled mobilization and differentiation of EPCs.^39^ These results rather clearly highlight the role played by EPCs in contributing to the DES-induced delayed arterial healing, with persistent fibrin deposition, sparse smooth muscle cell coverage, and incomplete re-endothelialization.^63^

### EPC-Coated Stents

In the DES era, concern for the use of cytostatic or cytotoxic drugs that produce a long-lasting inflammatory response, delayed endothelialization, and vasomotor dysfunction prompted the idea that a bioengineered DES with luminal surface covered with an anti-CD34^+^ antibody able to capture EPCs might promote a “controlled” healing.^64^ Upon stent placement, the anti-human CD34 antibodies would therefore attract circulating EPCs and promote a rapid stent re-endothelialization. This accelerated healing might translate into a decreased risk of stent thrombosis and restenosis, with the potential benefit to reduce the duration of dual antiplatelet therapy (DAPT).^50^ A representative EPC-capturing stent is the Genous stent (OrbusNeich, Ft. Lauderdale, FL), which uses monoclonal antibodies against CD34. The safety and efficacy of this novel stent were shown by preliminary investigations.^65,66^ Unfortunately, the Genous stent has shown a trend toward higher rates of target vessel failure in the TRI-stent Adjudication Study-High risk of Restenosis (TRIAS-HR) study,^67^ as well as in the Healthy Endothelial Accelerated Lining Inhibits Neointimal Growth First-in-Man (HEALING) study and the HEALING II study.^68^ Similar poor results have also been obtained with CD-133-coated coronary stents, which have been tested in a porcine model.^69^ With this background, the EPC-capturing technology was applied to a commercially available sirolimus-eluting stent to minimize the hyperproliferative reaction to the damaged vessel wall and suppress late loss.^70^ This led to the development of a specifically engineered device, the Combo (OrbusNeich Medical, Ft. Lauderdale, FL), which combines sirolimus elution from an abluminal biodegradable polymer matrix along with a covalently bound CD34 antibody layer, designed to control neointimal proliferation and to promote vessel healing with an accelerated stent strut tissue coverage. The Randomized study to Evaluate the safety and effectiveness of an abluMinal sirolimus coatED bio-Engineered StEnt (REMEDEE) study was the first trial comparing Combo stents to the paclitaxel DES (Taxus Liberte, Boston Scientific, Marlborough, MA).^71^ An optical coherence tomography subanalysis documented a higher percentage of uncovered struts per stent with the Combo than the Taxus Liberte at 60 days.^72^ Anyway, at the 9-month follow-up, the Combo stent was found to be noninferior to Taxus Liberte and the overall rate of clinical events was similar in the 2 groups, with no stent thrombosis up to 12 months.^71^ The Japan-USA Harmonized Assessment by Randomized, Multi-Center Study of OrbusNEich’s Combo StEnt (HARMONEE) study demonstrated noninferiority of the Combo stent to Xience (Abbott Vascular, Santa Clara, CA), although the trend toward a higher target vessel failure at 12 months was mainly driven by ischemia-driven target vessel revascularization, as stent thrombosis was detected only in one patient who received a Xience stent.^73^ The HARMONEE trial raised the suspicion that the endoluminal CD34 antibody layer could portend an actually increased—rather than decreased—hyperplastic reaction, but we have to acknowledge the substantial difformities of the platforms being compared, as the Combo struts were thicker (100 µm) than in the Xience stent (81 µm). In the REMEDEE trial, where the thickness of the comparator Taxus Liberte was similar (97 µm), the angiographic in-stent late lumen loss was similar (0.39 ± 0.45 mm vs. 0.44 ± 0.56 mm). The favorable results of randomized clinical trials have been recently confirmed in large registries .^74-77^

A major advantage of using the Combo stent might be the short duration of DAPT. The safety and efficacy of 3 months of DAPT after Combo stenting in patients with an ACS have been evaluated in the Randomised Evaluation of short-term DUal antiplatelet therapy in patients with ACS treated with the COMBO dual-therapy stEnt (REDUCE) trial, showing noninferiority to standard 12-month DAPT duration.^78^

Recently, results of the SORT OUT X trial, a randomized, multicenter, single-blind, trial with registry-based follow-up were reported. Overall, 3146 patients were randomized to treatment with the DTS (1578 patients) or with the sirolimus-eluting Orsiro stent (Biotronik, Bülach, Switzerland) (SES) (1568 patients). At 12 months, intention-to-treat analysis showed that rates of death, cardiac death, and myocardial infarction at 12 months did not differ significantly between the 2 stent groups. However, the SES was superior to the DTS mainly because the DTS was associated with an increased risk of target lesion revascularization. Differences in the DES technologies between the study stents might well explain these results, as the 2 stents have different drug-eluting kinetics and different strut thickness (100 μm for Combo and 60-80 μm for Orsiro).^79^

## EPCs and Cardiovascular Outcome

### EPCs and Outcomes in Stable Patients

**Figure 5.**
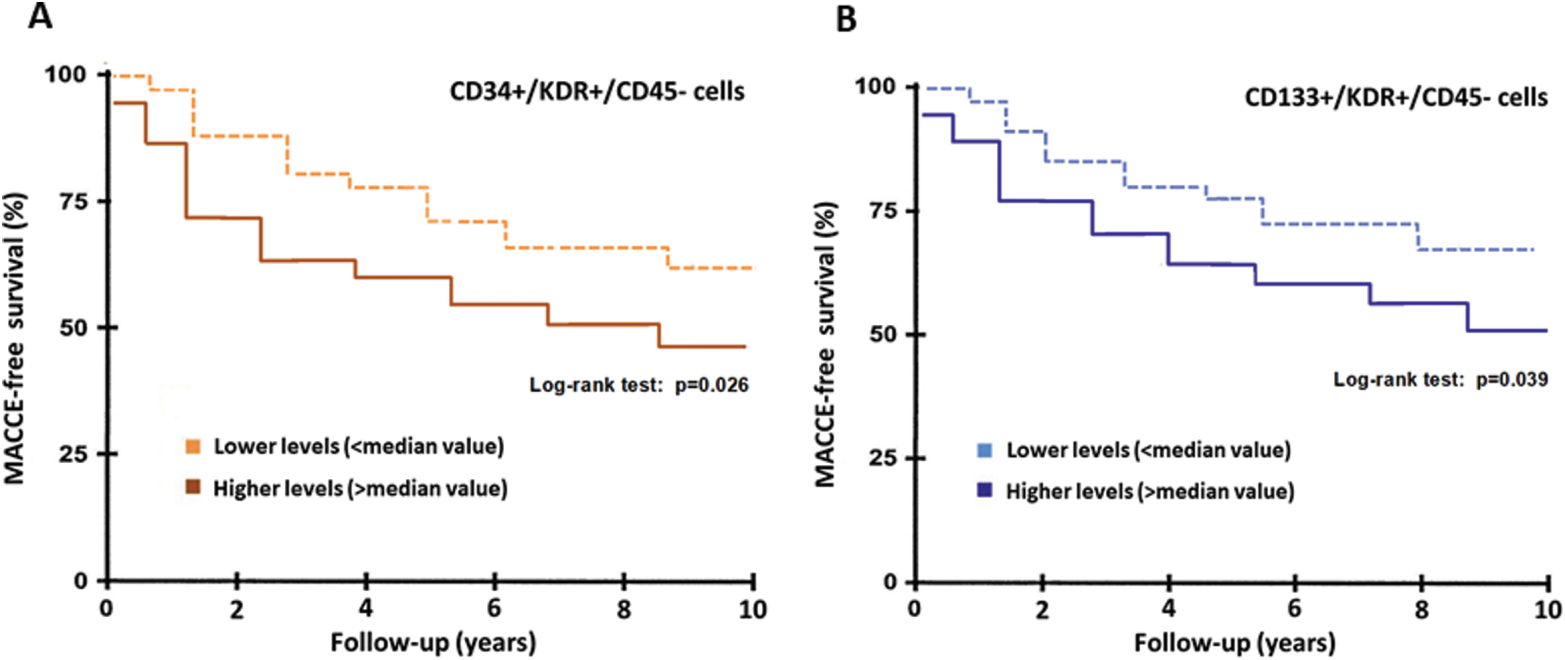
Survival curves at 10 years in patients with high versus low EPCs counts. Kaplan-Meier 5-year survival curves (MACCE-free) of patients stratified according to (**A**) quantity of CD34^+^/KDR^+^/CD45^−^ cells lower or higher than the median value and (**B**) quantity of CD133^+^/KDR^+^/CD45^−^ cells lower or higher than the median value. Modified from Pelliccia et al.^83^ EPCs, endothelial progenitor cells; KDR, kinase insert domain receptor.

EPCs have been consistently associated with cardiovascular outcome and death in stable patients with angiographic evidence of CAD (Supplementary Table 3).^30,35,36,43,58,80-83^ Overall, abnormal baseline EPC counts are associated with an approximately 2-fold increased risk of major adverse cardiovascular events. In the pivotal work by Werner et al, lower levels of CD34^+^KDR^+^ cells related to the short-term risk of cardiovascular death.^30^ In subsequent investigations, conversely, higher EPCs counts at baseline have been associated with an unfavorable long-term outcome in stable angina patients treated with PCI (Fig. 5).^83^ Thus, a sustained release of circulating EPCs might be considered a defense mechanism of the vessels in an attempt to compensate for more aggressive pathogenetic factors of atherosclerosis.^83^

### EPCs and Outcome in ACS

EPCs have recently been shown to be also a major marker for predicting the outcome of ACS (Supplementary Table 3).^39,46,84^ Samman Tahhan et al studied 2028 patients with unstable angina, AMI, or stable CAD, and found that numbers of circulating CD34^+^ cells (specifically, CD34^+^, CD34^+^/CD133^+^, CD34^+^/CXCR4^+^, and CD34^+^/VEGF cells) were higher in patients with AMI as compared to those with unstable angina or stable CAD.^46^ Among patients with ACS, higher EPC counts were associated with lower mortality, thus confirming the potential role of EPCs in myocardial repair processes and showing a novel role for precursor cells in predicting outcomes in patients with ACS.^8^

### EPCs as Biomarkers of Cardiovascular Disease

Besides their pathophysiological implications, EPCs have been extensively studied as a novel prototype of cardiovascular risk biomarkers.^84^ At present, biomarker-guided risk factor characterization of patients with CAD is based on various hematologic parameters, that is, leukocyte subpopulations (neutrophils and monocytes) and inflammatory indices (eg, C-reactive protein), that are not necessarily involved in the underlying pathologic processes. Although the mechanisms of action of EPCs are still not fully elucidated, there is mounting evidence that these progenitor cells might significantly improve the long-term stratification of cardiac patients. A patient-level analysis of data from 5 longitudinal studies found that baseline circulating progenitor cell count, including EPCs, improved discrimination of patients with a future cardiovascular event when added to a standard risk assessment model.^85^ Of note, the addition of progenitor cell count to a fully adjusted risk model including C-reactive protein allowed a better reclassification of up to 20% of patients into the appropriate risk category model.^85^ More recently, a combination of multiple plasma biomarkers centered around tissue remodeling, inflammation, renal dysfunction, and liver fibrosis has shown to be highly predictive of cardiovascular outcome.^86^ Further research is needed to confirm if a multimarker approach including EPCs count and coupled with machine-learning might represent a promising strategy for enhancing risk stratification in CAD.

## Concluding Remarks

In summary, current data indicate that EPCs are not innocent bystanders, but active players in maintaining a healthy coronary anatomy and function. The theoretical benefit of EPC-capture DES, which is currently documented only in terms of safety, will likely emerge when the new technology will be available on DES with thin struts. Also, evidence now exists that the measure of circulating EPCs might improve risk stratification beyond traditional assessment model.^87^ Last but not least, EPCs have also been studied as candidate cell sources for cardiovascular regeneration, and results of several preclinical and clinical studies addressing their therapeutic potential in patients with CAD have been comprehensively discussed in previous reviews.^88, 89^

In conclusion, available scientific information supports the clinical utility of EPCs, which should now be incorporated into clinical practice for more precisely predicting individual risk of long-term major adverse cardiovascular events.

## Supplementary Material

szac010_suppl_Supplementary_Materials

## Data Availability

The data that support the findings of this study are available from the corresponding author upon reasonable request.
