# Supplementary material for: Endothelial Progenitor Cells in Coronary Artery Disease: From Bench to Bedside
Source: Stem Cells Transl Med. 2022 Apr 1;11(5):451–60. doi: 10.1093/stcltm/szac010 (PMC9154346; doi:10.1093/stcltm/szac010)
Supplement: szac010_suppl_Supplementary_Materials [file szac010_suppl_supplementary_materials.pdf]

SUPPLEMENTAL INFORMATION

Search strategy

Review of the available literature on endothelial progenitor cells (EPCs) was conducted following current guidelines, including the Cochrane Collaboration and Meta-analysis Of Observational Studies in Epidemiology (MOOSE)(S1), and the Preferred Reporting Items for Systematic reviews and Meta-Analyses (PRISMA) protocols amendment to the Quality of Reporting of Meta-analyses (QUOROM) statement (S2).

The Pubmed, Scopus, and Web of Science databases were searched up to June 30, 2021. Search key-words included "acute coronary syndrome", "atherosclerosis", "anti-CD34+", "biomarkers", "cardiac death", "Combo^{TM}", "coronary artery disease", "coronary atherosclerosis", "drug-eluting stent", "dual-therapy stent", "endothelium", "endothelial cells", "endothelial progenitor cells", "in-stent restenosis", "myocardial infarction", "outcome", "percutaneous coronary intervention", "prognosis", "restenosis", "stem cells". Initial findings were supplemented with manual searches of the bibliographies of relevant papers. No language restriction was enforced in order to minimize the risk of publication bias.

The study inclusion criteria were investigations that assessed the association of EPCs count with major components of coronary artery disease, including angiographic evidence of coronary atherosclerosis, restenosis following percutaneous coronary intervention, and cardiovascular outcomes either in stable patients or in patients with acute coronary syndromes.

The study exclusion criteria included duplicate reporting, in which case the manuscript reporting the largest sample of patients was selected, or, if equal, the study with the largest number of overall patients.

Retrieved citations were first screened independently by two unblinded investigators (FP and MZ). Studies identified as potentially relevant on the basis of title or abstract were selected for full review. The reviewers independently assessed these investigations for eligibility and disagreement was resolved by consensus. Data from each study were then extracted and entered into a structured spreadsheet.

Supplemental references

1. Moher D, Cook DJ, Eastwood S, Olkin I, Rennie D, Stroup DF. Improving the quality of reports of meta-analyses of randomised controlled trials: the QUOROM statement. Quality of reporting of meta-analyses. Lancet 1999; 354:1896-900.

2. Altman DG, Tetzlaff J, Mulrow C, et al. The PRISMA statement for reporting systematic reviews and meta-analyses of studies that evaluate healthcare interventions: explanation and elaboration. BMJ 2009; 339:b2700.

3. von Elm E, Altman DG, Egger M, Pocock SJ, Gøtzsche PC, Vandenbroucke JP; STROBE Initiative**.** The Strengthening the Reporting of Observational Studies in Epidemiology (STROBE) statement: guidelines for reporting observational studies**.** Lancet 2007;370:1453-7*.*

Supplemental table 1. Summary of observational studies evaluating the association of EPCs counts with angiographic evidence of coronary atherosclerosis

First Author (Ref.)	Country	Year	Patients (n)	EPCs subtypes	Main findings
Vasa (27)	Germany	2001	15	$CD34^+$/ KDR+	$CD34^+$/ KDR+ lower in patients with CAD compared to controls
Eizawa (28)	Japan	2004	34	$CD34^+$	$CD34^+$ significantly reduced (-30%) in patients with CAD compared to controls
Guven (29)	US	2006	48	$CD34^+$/ KDR+	EPCs increased in patients with significant CAD as compared to patients without significant CAD
Werner (30)	Germany	2007	90	$CD34^+$/KDR^+<br>$CD133^+$/ KDR+	Low EPC numbers correlated with severety of CAD
Wang (31)	China	2007	60	$CD34^+$/ KDR+	$CD34^+$ significantly lower in patients with severe CAD compared to mild CAD
Ripa (32)	Denmark	2007	54	$CD34^+$/ KDR+	CD34+ slilghtly increased in patients with CAD compared to controls
Liguori (33)	Italy	2008	40	$CD34^+$	$CD34^+$ significantly reduced in patients with CAD as compared with controls
Pelliccia (34)	Italy	2010	105	$CD34^+$/KDR^+<br>$CD133^+$/ KDR+	EPCs similar in patients with significant CAD and controls
Padfield (35)	UK	2013	201	$CD34^+$/KDR^+<br>$CD133^+$/ KDR+	EPCs not increased in patients with ACS, and unrelated to CAD severity or clinical outcome

Hayek (36)	US	2016	1497	$CD34^+$/ KDR+	CD34+ and CD34+/ KDR+ significantly lower in patients with CAD and PAD as compared with CAD only

CAD= Coronary artery disease; EPCs= Endothelial progenitor cells; PAD=Peripheral artery disease.

Supplemental table 2. Summary of observational studies evaluating the association of EPCs counts with restenosis after PCI

First Author (Ref.)	Country	Year	Patients (n)	Procedure Stent	EPCs subtypes	Main findings
Schober (56)	Germany	2005	17	Elective PCI BMS only	$CD34^+$	Post-procedural CD34+ cell counts increased in patients with restenosis but decreased in patients without restenosis
Inoue (57)	Japan	2007	30	Elective PCI BMS only	$CD34^+$	CD34+ cells increased from day 7 to day 14 after PCI and was more striking in patients with restenosis
Pelliccia (34)	Italy	2010	105	Elective PCI BMS only	CD34+/KDR+/CD45- CD133+/ KDR+/CD45-	EPCs significantly higher in patients who experienced restenosis as compared to stable CAD
Briguori (58)	Italy	2010	136	Elective PCI DES (40% cases)	$CD34^+$/ KDR+	Significant inverse relationship between EPCs at baseline and restenosis at 24-month
Bonello (53)	France	2012	156	Elective PCI BMS only	CD34+KDR+	EPCs levels at baseline, 6-h and 24-h similar between patients with and without restenosis
Wu (59)	Taiwan	2014	130	Elective PCI Unknown	$CD34^+$/KDR^+ $CD133^+$/ KDR+	Lower levels of EPCs count associated with the highest restenosis rates
Haine (60)	Belgium	2014	124	Elective PCI BMS only	CD34+ KDR+	EPCs count before PCI do not predict coronary in-stent restenosis
De Maria (61)	Italy	2015	20	Elective PCI	CD34+/KDR+/CD45-	Increased EPC levels associated with greater

				BMS/DCB (50%)		neointimal hyperplasia and uncovered stent struts
Otto (39)	Germany	2017	43	Elective PCI DES only	$CD34^+/KDR^+$ $CD133^+$/ KDR+	Increased EPC levels associated with less sub-stent coronary plaque burden
Montenegro (52)	Brazil	2018	37	Elective PCI DES (27% cases)	CD34+ CD133+	No significant association between EPC and restenosis

CAD=Coronary artery disease; EPCs=Endothelial progenitor cells; PCI=Percutaneous coronary intervention.

Supplemental table 3. Summary of observational studies evaluating the association of EPCs counts with cardiovascular outcomes either in stable patients or in patients with acute coronary syndromes.

First Author (Ref.)	Country	Year	Patients (n)	Patients' characteristics	EPCs subtypes	Follow-up (months)	Main findings
**STUDIES IN PATIENTS WITH STABLE CONDITIONS**							
Werner (30)	Germany	2005	507	Coronary angiography	$CD34^+$/ KDR+	12	Lower levels of EPCs associated with a higher risk of death from cardiovascular causes
Briguori (58)	Italy	2010	136	Elective PCI	$CD34^+$/ KDR+	24	Lower levels of EPCs associated with a higher MACE rate after PCI
Padfield (35)	UK	2013	201	Coronary angiography	CD34+/CD45-	36	Increased EPCs predict future cardiovascular events
Chiang (80)	Taiwan	2014	77	Elective PCI	CD34+ KDR+ $CD133^+$/ KDR+	48	Lower levels of EPCs associated with a higher MACE rate after PCI
Patel (81)	UK	2015	905	Coronary angiography	CD34+ KDR+ $CD34^+/CD133^+$	22	Reduced circulating EPCs associated with risk of death
Hayek (36)	US	2016	1,497	Coronary angiography	$CD34^+$/ KDR+	24	EPCs significantly lower in patients with increased risk of mortality
Hammadah (43)	US	2017	566	Coronary	$CD34^+$	36	Low CD34+ levels

				angiography			predicted adverse cardiovascular outcomes
Mehta (82)	US	2020	1,281	Coronary angiography	CD34+,CD34+/CD13 +, CD34+/KDR+	42	Low CD34+ levels associated with a higher myocardial infarction and mortality
Pelliccia (83)	Italy	2020	149	Elective PCI	CD34+/KDR+/CD45- CD133+/ KDR+/CD45-	120	Higher levels of EPCs associated with a higher MACE rate after PCI
**STUDIES IN PATIENTS WITH ACUTE CORONARY SYNDROME**							
Schmidt-Lucke (38)	Germany	2005	77	ACS	CD34^{+}/ KDR+	10	Reduced numbers of EPCs associated with a significantly higher incidence of cardiovascular events
Cuadrado-Godia (84)	Spain	2015	100	AMI	CD34^{+}/CD133^{+}/ KDR+	6	Low EPC count associated with new cardiovascular events
Samman Tahhan (46)	US	2018	529	ACS	CD34^{+}/ KDR+	24	EPCs levels significantly higher in patients after an AMI compared to stable CAD. In ACS, a lower number of EPCs associated with a higher mortality

ACS= Acute coronary syndrome; AMI=Acute myocardial infarction; CAD= Coronary artery disease; EPCs= Endothelial progenitor cells; MACE=Major adverse coronary events; PCI=Percutaneous coronary intervention.
